# Draft Genome Sequences of *Geobacillus* sp. Strains CAMR5420 and CAMR12739

**DOI:** 10.1128/genomeA.00567-14

**Published:** 2014-06-05

**Authors:** Pieter De Maayer, Carolyn E. Williamson, Christopher T. Vennard, Michael J. Danson, Don A. Cowan

**Affiliations:** a Centre for Microbial Ecology and Genomics, Genomics Research Institute, Department of Genetics, University of Pretoria, Pretoria, South Africa; b Centre for Extremophile Research, Department of Biology and Biochemistry, University of Bath, Bath, United Kingdom

## Abstract

Thermophilic *Geobacillus* spp. can efficiently hydrolyze hemicellulose polymers and are therefore of interest in biotechnological applications. Here we report the genome sequences of two hemicellulolytic strains, *Geobacillus* sp. CAMR12739 and CAMR5420.

## GENOME ANNOUNCEMENT

Members of the genus *Geobacillus* are Gram-positive, thermophilic, spore-forming, aerobic bacteria (1). They have an extensive capacity to degrade plant cell wall hemicellulose polymers into their component pentose sugars, a capacity that has been linked to a single chromosomal locus (2, 3). Coupled with this is their production of thermostable hydrolytic enzymes, resulting in considerable interest in the use of *Geobacillus* in a range of biotechnological applications (4). *Geobacillus* sp. strains CAMR5420 and CAMR12739 were acquired from the CAMR (Porton Down, United Kingdom) thermophile culture collection, which is now held by the Centre for Extremophile Research, Department of Biology and Biochemistry, at the University of Bath, United Kingdom. CAMR5420 was added to the collection in 1989; CAMR12739 was collected in Skaltholt, Iceland, and deposited in the collection in 1987.

Genome sequencing was performed using the 454 GS-FLX (CAMR12739) and Illumina GAIIx (CAMR5420) platforms, yielding 2,533,592 reads (average read length of 138 nucleotides [nt]) for CAMR53420 and 119,419 reads (average read length of 365 nt) for CAMR12739. Assembly was performed using a combination of the CLC Genomics Workbench v6, DNAstar Ngen and Velvet v1.1 (5) assemblers. Finally, reference assembly was undertaken using the complete genomes of related *Geobacillus* spp. using Mauve (6). The genome of CAMR5420 was assembled into 96 contigs, yielding a total genome size of 3.50 megabases, with a mean G+C content of 51.89%. The genome of CAMR12739 was assembled into 74 contigs, with a total genome size of 3.41 Mb and a mean G+C content of 52.21%. Further *in silico* assembly was hampered by the presence of highly conserved transposase genes. The genomes were annotated using the Rapid Annotations using Subsystems Technology (RAST) server (7). The genomes of CAMR5420 and CAMR12739 code for 3,816 and 5,836 proteins, respectively. A single, but variable, locus underlying the capacity of these strains to hydrolyze and utilize hemicellulose polymers was identified. The genomes of *Geobacillus* sp. CAMR5420 and CAMR12739 will be employed for comparative genomic analyses, with the goal of developing recombinant *Geobacillus* strains with optimal hemicellulose degrading capacities.

### Nucleotide sequence accession numbers.

These whole-genome shotgun projects have been deposited at DDBJ/ENA/GenBank under the accession no. JHUS00000000 (CAMR5420) and JHUR00000000 (CAMR12739). The versions described in this paper are the first versions, JHUS01000000 (CAMR5420) and JHUR01000000 (CAMR12739).
